# Authors’ Reply – Comments: Serum levels of infliximab in Brazilian patients with Crohn's disease: what are the reasons for differences from previous studies?

**DOI:** 10.6061/clinics/2019/e1517

**Published:** 2019-09-19

**Authors:** Luis Eduardo Miani Gomes, Francesca Aparecida Ramos da Silva, Lívia Bitencourt Pascoal, Renato Lazarin Ricci, Guilherme Nogueira, Michel Gardere Camargo, Maria de Lourdes Setsuko Ayrizono, João José Fagundes, Raquel Franco Leal

**Affiliations:** I Laboratorio de Investigacao em Doencas Inflamatorias Intestinais, Servico de Coloproctologia, Faculdade de Ciencias Medicas, Universidade Estadual de Campinas (UNICAMP), Campinas, SP, BR; II Laboratorio de Sinalizacao Celular, Universidade Estadual de Campinas (UNICAMP), Campinas, SP, BR

To the Editor:

We would like to thank Teixeira et al. 1 for their comments on our article 2. They correctly noted that the majority of our patients presented infliximab (IFX) levels above the therapeutic concentration, differing from three other Brazilian studies published between 2017 and 2018 3-5. However, there are several significant conceptual and methodological differences between those studies and ours that need to be taken into consideration to understand the factors that led to such contrasting results. Our aim was to perform a prospective observational study, not an interventional one. Nor did we aim to establish the best minimum and maximum cut offs of the therapeutic levels of IFX for Brazilian Crohn's disease (CD) patients.

In 2017, Kampa et al. 3 conducted the first Brazilian investigation in this field (reference 10 of the Gomes et al. Manuscript), which measured IFX levels and anti-infliximab antibodies (ATI). However, Kampa et al. themselves admitted that the limitations of their paper were its retrospective character (failures in data collection might occur) and the absence of a detailed description of the clinical and endoscopic conditions at the moment of collection. Additionally, the aim of Teixeira et al. 4 was to measure the serum levels of IFX by comparing two different, already validated, assays 6,7. Although this approach was indeed interesting, the majority of patients included in the study (88%) were in remission, a condition that is not impacted by therapeutic drug monitoring in clinical practice. Teixeira et al. 4 even suggested that more studies on patients with active disease would be necessary. Finally, Parra et al. 5 evaluated the levels of IFX in a larger cohort of 55 patients with CD and 16 with UC. The novelty of this study was the use of a quality of life questionnaire and its correlation with the serum trough levels of IFX. Although they did perform their analysis considering patients in a state of disease activity or remission, the criterion for disease activity was based on an elective colonoscopy examination performed at least three months before and three months after blood collection. This interval can be deemed rather too long in the case of inflammatory bowel diseases. By contrast, we only took into consideration the interval of up to one month before or after sample collection. Moreover, as acknowledged by Parra et al. 5 themselves, they did not include variables for either proinflammatory biomarkers (such as CRP and fecal calprotectin) or body mass index (BMI) in their analysis. We assessed the serum albumin levels of all patients included in our research.

All of the studies cited above performed analyses that included patients with CD and ulcerative colitis (UC). Our study included only CD patients because the molecular characteristics of each disease may differ in response to a specific drug. For histological healing, for example, the IFX trough level requirement for CD cases is ≥9.8 µg/mL, whereas it is ≥10.5 mg/L for UC patients 8,9.

Our study is the first Brazilian prospective observational study to measure the levels of IFX and ATIs in CD patients, assessing disease activity by colonoscopy or nuclear magnetic resonance (NMR), including in the analysis at least one nutritional parameter (albumin level) that is relevant to the drug pharmacokinetics 10 (reference 27 of the Gomes et al. manuscript). Therefore, it should not at all come as a surprise that patients with a relatively favorable nutritional parameter, such as albumin, present adequate or supratherapeutic levels of IFX in both groups (active and in remission). Recently, we published a paper regarding the nutritional aspects of 60 CD patients receiving treatment at our institution 11. Although relevant differences were observed in nutritional markers for patients in remission and with active disease, the prealbumin serum levels did not differ between these groups. We emphasize the need for a multidisciplinary team, including nutritional follow-up, so that these patients can achieve better outcomes.

Regarding the therapeutic window noted by Teixeira et al. 1, Ungar et al. 12 found a significant association between anti-TNF-α serum levels and mucosal healing, which led to the recommendation that a serum level of 6-10 μg/mL for IFX is necessary to achieve healing of 80%-90% of IBD patients, which may be considered a therapeutic window as well. Moreover, this interval was followed in other studies in the literature 13-15. Some authors propose minimum levels of up to 10 µg/mL or even 18 μg/mL for fistula healing 12,14. Drobne et al. recently observed that maintaining higher infliximab levels >7 μg/mL provided better control of IBD without an increased risk of infection 13. Our choice for the quantitative ELISA from Promonitors (Progenika Biophama, S. A. Spain) was due to its widespread use in other institutions around the world and its greater availability for purchase in our location, in addition to the possibility to perform the ELISA test in our laboratory and to standardize the technique without the need to send samples abroad, which would involve high costs and additional approvals by national research ethical committees.

Concerning the comments of Teixeira et al. 1 about why we might have mixed the results of the study because several patients with levels between 3-6 µg/mL would be considered adequate according to the range of 3-7 µg/mL but infratherapeutic according to our adopted range (6-10 µg/mL), we would like to clarify that, as described in our manuscript 2, there were no patients with levels between 3-6 µg/mL. Of the few patients we had with IFX trough levels ≤ 5 µg/mL, all presented undetectable levels (<0.035 µg/mL), and they correlated 100% with positive ATIs, demonstrating the accuracy of the test.

Concerning the similar anti-TNFα levels that we observed in both the active disease and remission groups 2, other studies in the literature have already noted this phenomenon 16,17. Patients with active mucosal disease may present a higher rate of serum-to-tissue drug level mismatch compared to those in remission 17. This kind of analysis may be more relevant to explain the correlation between therapeutic drug monitoring and the presence of intestinal inflammation, rather than the simple isolated serum drug level. Low serum drug levels may not always correlate with patients in terms of disease activity because they may depend more on the ratio of serum and tissue levels than on only serum levels. Although there was no correlation with CD activity in our study, the IFX and ATIs levels correlated with each other (all patients with undetectable levels of IFX had positive or close to positive ATI levels), as described in our study. This scenario may be important in patients with active disease to improve their clinical management.

For all of these reasons, unfortunately, the previous Brazilian studies mentioned above are sufficiently different from ours to render them not at all comparable. However, the apparent discrepancy between our results and theirs indicates the need for further dialogue in the field concerning different fundamental approaches. Prospective studies that assess IFX and ATIs levels at more than one point in time 18 (reference 33 of the Gomes et al. manuscript), in addition to performing validated assays in our own Brazilian institutions, may bring the results closer to the reality of clinical practice. A prospective study analyzing IFX levels by two different assays (one of them a rapid test), and detecting ATIs levels is ongoing at our institution to better clarify our findings and to determine how much they may help in the management of CD.
